# The Hygiene of the Eyes

**Published:** 1877-11-20

**Authors:** 


### THE HYGEINE OF THE EYES.

The following hygienic rules are compiled and condensed from eminent French and English authorities:

For the worker the light should come as much as possible from the left side, that is to say, from the side towards which one turns in working. Daylight is the best; but direct sunlight and that reflected from mirrors should be avoided. The aspect should be northern, and the light should come a little from above.

White walls should be avoided; highly varnished tables and, in workshops, shining articles like silk should be protected from the sun's rays.

Artificial light is always bad, on account of the heat and the exhalation of carbonic acid. The best is that of lamps fed with vegetable oil (much used in France, but seldom in this country) and furnished with a glass shade. Gas is bad, because of its heat, brilliancy and mobility; the light of mineral oil is too hot; that of candles insufficient and flickering. The eye of the workman should avoid the light coming to him directly, or diffused through the room.

Working immediately after meals is objectionable; also uninterrupted use of the eyes for long periods of time. One should write on an inclined plane, and not keep the head bent down more than is absolutely necessary. Reading in bed is bad every way.

Some good authorities commend washing the eyes with cold water, but the majority of the best ophthalmologists advise the use of hot water for the less serious affections of the eye. For tired eyes, we believe, from our own experience, that water hot as can be borne is refreshing and beneficial.

If the eyes are fatigued by bad artificial illumination, blue or slightly smoked glasses will be useful, and in order to avoid the lateral rays they should be large and round.

If the irritation of the eyes persists, all work must be abandoned, and an examination made to see if there be any disturbance of refraction, of power of accommodation, or of the mobility of the eyes.

Presbyopia, or so-called "far-sightedness," supervenes earlier with those who are constantly at work than with other individuals, and as soon as it does convex glasses should be at once resorted to, without which the muscle of accommodation would be fatigued to no purpose. At first they should be used for working in the evening, after the fatigue of the day; but a long-sighted person should only use spectacles for looking at near objects, not at far ones.

Work requiring close application favors the development of myopia, or "near-sightedness," precisely in proportion as the conditions of illumination are bad. If the action of those causes continues, the myopia will increase until vision is lost.

A slight degree of myopia may be favorable to close work, but, as a general rule, work requiring close application, by the derangement of circulation that it inevitably induces in the eye, is much more injurious to the myopic, and is the great cause of the development of myopia and its complaints. Young people should be examined, and if they are myopic, hindered from undertaking tedious studies and all professions demanding close application of the eye.—*Boston Journal of Chemistry.*

